# Two-Dimensional and Point Shear-Wave Elastography to Predict Esophageal Varices and Clinically Significant Portal Hypertension in Patients with Chronic Liver Disease

**DOI:** 10.3390/jcm13247719

**Published:** 2024-12-18

**Authors:** Myriam W. Heilani, Max Bolender, Victoria T. Mücke, Katharina M. Schwarzkopf, Alica Kubesch-Grün, Nada Abedin, Georg Dultz, Stefan Zeuzem, Christoph Welsch, Mireen Friedrich-Rust, Jörg Bojunga, Eva Herrmann, Marcus M. Mücke

**Affiliations:** 1 Medical Clinic 1, University Hospital, Goethe University Frankfurt, 60590 Frankfurt am Main, Germany; heilani@med.uni-frankfurt.de (M.W.H.); max.bolender@sana.de (M.B.); v.muecke@med.uni-frankfurt.de (V.T.M.); schwarzkopf@med.uni-frankfurt.de (K.M.S.); alica.kubesch-gruen@unimedizin-ffm.de (A.K.-G.); nada.abedin@gmail.com (N.A.); dultz@em.uni-frankfurt.de (G.D.); zeuzem@em.uni-frankfurt.de (S.Z.); welsch@med.uni-frankfurt.de (C.W.); mireen.friedrich-rust@unimedizin-ffm.de (M.F.-R.); bojunga@med.uni-frankfurt.de (J.B.); 2 Institute of Biostatistics and Mathematical Modeling, Goethe University Frankfurt, 60590 Frankfurt am Main, Germany; herrmann@med.uni-frankfurt.de

**Keywords:** liver cirrhosis, complication of liver disease, non-invasive diagnostic, liver stiffness

## Abstract

**Introduction:** The non-invasive assessment of disease severity remains pivotal in patients with chronic liver disease (CLD) as it has wide implications in predicting liver-related complications or death. Shear-wave elastography (SWE) is an emerging ultrasound-based method to non-invasively measure liver stiffness. The aim of our study was to evaluate two-dimensional (2D) and point (p) SWE to predict the presence of esophageal varices (EV) or clinically significant portal hypertension (CSPH). **Methods:** This was a retrospective analysis of a prospectively performed cohort study of patients with CLD treated in the outpatient clinic of the Frankfurt University Hospital. PSWE using the Hitachi HI Vision ASCENDUS system and the Siemens ACUSON S2000^TM^ system or 2D-SWE using the Toshiba APLIO500 system were analyzed at baseline and during follow-up to predict EV or surrogate parameters of CSPH. ROC curves were calculated for pooled liver stiffness measurements (LSMs) using a bootstrap approach. A combined model of SWE and platelet count was created and a mixed-effect logistic regression analysis using log-transformed values was performed. **Results:** Overall, 511 patients with CLD and 919 consecutive LSMs were included and 315 patients (61.6%) had signs of CSPH. 2D-SWE performed best to predict EV and CSPH, and the addition of platelet count to the predictive model significantly increased test results for EV (AUC 0.83, 95%-CI: 0.76–0.89; difference in AUC 0.11, 95%-CI: 0.03–0.19, *p* = 0.004), but only marginally for CSPH (AUC 0.75, 95%-CI: 0.64–0.85; difference in AUC 0.06, 95%-CI: 0.02–0.14, *p* = 0.150). LSM > 18.5 and >20 kPa were indicative of CSPH and EV, while LSM < 10 kPa and <11 kPa ruled out CSPH and EV, respectively. **Conclusions:** Our study found that 2D-SWE in combination with platelet count performed best (in comparison to the other SWE methods) to predict EV or CSPH in patients with CLD. Future prospective trials are needed to validate our results.

## 1. Introduction

Chronic liver disease (CLD) represents a major global health threat, and it may be caused by chronic viral hepatitis, chronic alcohol misuse, metabolic, autoimmune, cholestatic, or hereditary disorders [1,2]. Chronic liver damage and inflammation lead to liver fibrosis and advanced chronic liver disease (aCLD), which is associated with portal hypertension, decompensation events, liver failure, and hepatocellular carcinoma [3]. Worldwide, more than two million deaths are estimated to be caused by CLD annually, mainly attributed to complication of liver cirrhosis [1].

At the time of diagnosis as well as during follow-up, a precise and adequate evaluation of the extent of fibrosis and stage of liver disease is warranted. Such information may influence the choice of treatment or the timepoint of therapy initiation [4,5,6] and provide invaluable information on the risk of disease progression and development of complications such as variceal bleeding, hepatic encephalopathy, ascites, or infection [3,7,8].

Nowadays, non-invasive tools such as serum markers and/or ultrasound-based elastography methods that can be repetitively performed have replaced routine liver biopsy in daily clinical practice, which is reserved for selected cases [3,6]. Vibration-controlled transient elastography (VCTE) has been extensively evaluated in patients with CLD and is one of the current standards to assess liver fibrosis and evaluate the risk of hepatic complications [7,8,9,10,11]. Disadvantages of VCTE include its higher failure rates in obese patients and in those with ascites [9]. Moreover, it is not integrated in an ultrasound system, and a separate device must be acquired in order to use it [9]. Liver stiffness measurements (LSM) using shear-wave elastography (SWE)—integrated in available ultrasound scanners—was a more recent addition to the armamentarium of non-invasive liver fibrosis assessment [12,13,14,15]. Using SWE, an acoustic radiation force generates tissue displacement on a micrometer level, that generates perpendicular shear waves propagating through the liver tissue [16,17]. By measuring the propagation speed of these shear waves, the tissue stiffness can be estimated. SWE can be classified as point (p) SWE if the acoustic radiation force is applied in one location, or as two-dimensional (2D) SWE if it is applied in multiple adjacent locations [17,18,19]. SWE has been proven to reliably rate stages of liver fibrosis [20,21], and predict complications and survival in (advanced) CLD in clinical studies [13,15,22,23,24]. However, it has not (yet) been comprehensively implemented in daily clinical routine. Diagnostic algorithms concerning SWE such as the Baveno VII criteria, which assess the risk of CSPH using VCTE and platelet counts, are scarce [25,26,27].

The aim of this study was to evaluate 2D-SWE and pSWE-based LSM to predict the presence of esophageal varices (EV) or surrogate parameters of clinically significant portal hypertension (CSPH) in patients with CLD.

## 2. Materials and Methods

### 2.1. Study Design

This was a retrospective analysis of a prospective longitudinal study of patients with CLD with different etiologies who were treated from July 2016 to August 2018 at the outpatient clinic of the Department of Internal Medicine I, Frankfurt University Hospital, Germany (see also Supplementary Figure S1). For the original study, patients were eligible for inclusion if they were adults (≥18 years) and diagnosed with a CLD. Patients suffering from hepatocellular carcinoma (HCC) outside Milan or any other malignancy, patients under immunosuppression or infected by the human immunodeficiency virus, patients with transjugular portosystemic shunt, and pregnant or breastfeeding women were excluded. Following enrollment, patient data were collected and stored in an electronic patient data collection form at baseline and during follow-up visits. Patients were followed on each readmission in the outpatients’ clinic. Additionally, at least one complete and valid data set of non-invasive LSM by SWE using the Hitachi HI Vision ASCENDUS system (Hitachi Medical Corporation, Chiyoda, Japan; abbreviation for this study: pSWE, point shear-wave elastography), the Siemens ACUSON S2000^TM^ system (Siemens AG, München, Germany; abbreviation for this study: acoustic radiation force impulse imaging, ARFI), or the Toshiba APLIO500 system (Toshiba Medical Systems Corporation, Otawara, Japan; abbreviation for this study: two-dimensional SWE, 2D-SWE) was required for the present study. For each SWE data set, at least 10 measurements were obtained, and median values were calculated according to the manufacturers’ instruction. Measurements were performed in the right lobe of the liver, 2–3 cm below the liver capsule in the absence of large vessels and bile ducts, through the intercostal space, after a minimum of 6 h of fasting on the same day. Invalid data were defined as fewer than 10 valid measurements, a success rate of <60%, or an interquartile range of ≥30%. Details of the different SWE methods and presets can be found in the Supplementary File and Supplementary Figure S2.

The performance of SWE to predict EV in patients with chronic liver disease or the presence of CSPH was evaluated and compared with the FIB-4 Score, a laboratory value-based score to screen for liver fibrosis [2]. In the first model (prediction of EV), all patients regardless of past or present decompensation events were allowed, and EV were diagnosed via endoscopy (documented within 24 months—median 273 days—from baseline; in patients with an endoscopy > 24 months from baseline or missing endoscopy data on the platelet count/spleen diameter ratio with a cut-off of 909 was additionally used to avoid misclassification; the platelet count/spleen diameter ratio with a cut-off of 909 is a parameter shown to be very robust and reliable to predict varices) [28]. The presence of CSPH was defined as either having relevant signs of portal hypertension in endoscopy (esophageal or fundus varices, or portal hypertensive gastropathy) or documented surrogate parameters of CSPH derived from a combination of clinical and imaging features (portocaval anastomosis, splenomegaly, ascites, or varices on imaging) as judged by the treating physician, leading to the prescription of non-selective beta-blockers (NSBB). Clinical data, ultrasound examination, and laboratory results were from the same day when the SWE was performed.

In the second model (prediction of CSPH), patients with past or present decompensation were excluded, as a screening for CSPH via 2D-SWE or any other modality would not be reasonably performed in daily clinical routine at that stage of the disease (apparent CSPH).

The study was performed in accordance with the Declaration of Helsinki. All patients had signed written informed consent before study inclusion. The local ethics committee approved this study (No. 2023-1573, 7 December 2023).

### 2.2. Statistical Analysis

Statistical analyses were performed with BiAS, version 11.03, and R using the lme4, pROC, and the rms packages. For patients’ characteristics, group differences were assessed by the Mann–Whitney U test and Fisher’s exact test for continuous or categorical variables, respectively.

The situation with repeated SWE assessments in a single patient required modifications of the standard statistical approach. Typically, patients were evaluated up to three times with the same LSM modality within one year (2D-SWE n = 143 patients with 229 consecutive LSMs, ARFI n = 255 patients with 527 consecutive LSMs and pSWE n = 113 patients with 163 consecutive LSMs). Therefore, sensitivities and specificities according to the analyzed outcome (EV and surrogate parameters of CSPH) at baseline, FU 6 months or FU 12 months were calculated. Additionally, a linear interpolation of sensitivity and specificity was used for all potential cut-off values and the mean sensitivity and specificity were calculated over up to three assessments. In order to evaluate the variability of this approach, receiver operating characteristic (ROC) curves were calculated in 1000 bootstrap samples over the patient data set and, finally, median values as well as confidence intervals (CI) for the different performance parameters (area under the curve, AUC, sensitivity, specificity, positive predictive value, PPV, and negative predictive value, NPV) were calculated from all bootstrap samples. For the combined model (platelet count and LSM using the 2D-SWE), the underlying mixed-effect logistic regression model used log-transformed values, and was calculated for each bootstrap sample. In this way, the over-optimistic assessment of the combined predictor could be reduced.

In addition, a graphical representation of the LSM was created via boxplots and the summarized ROC curve over the bootstrap samples together with single ROC curves of 100 bootstrap samples for each assessment method. The final prediction model based on the combination of 2D-SWE measurements and platelet counts is represented with a nomogram which used the fixed effects from the mixed-effect logistic regression model.

## 3. Results

### 3.1. Patient Characteristics

Overall, 511 patients with CLD were included in this study, and patients’ characteristics are displayed in Table 1. More than half of the patients (57.9%) were male and had a mean age of 59 years (range of 27–89 years). The underlying etiology of CLD was viral hepatitis (35.4%), alcoholic liver disease (ALD, 24.3%), and metabolic dysfunction-associated steatotic liver disease (MASLD, 17.4%) in the majority of cases. 286 patients (56.0%) had no previous episodes of decompensation and the mean MELD Score and Child–Pugh Score were 10 and 6, respectively. The mean SWE values obtained were 2.66 m/s (±0.97 m/s) with ARFI (Siemens ACUSON), 11.31 kPa (±4.68 kPa) with pSWE (Hitachi VISION), and 16.30 kPa (±9.10 kPa) with 2D-SWE (Toshiba APLIO500). 315 patients (61.6%) showed signs of CSPH. Accordingly, the Child–Pugh Score, FIB-4 Score, serum bilirubin, and international normalized ratio (INR) were significantly higher, and the serum albumin and platelet counts were significantly lower in the group of patients with CSPH (*p* < 0.001 for all comparisons). In the patients with CSPH, ascites was present in 79 patients (25.1%) and 239 patients (75.9%) had esophageal varices upon endoscopy. Significantly more patients with ALD (*p* < 0.001) and fewer patients with viral hepatitis (*p* = 0.002) presented with signs of CSPH at study inclusion. If patients with ascites or a history of decompensation were excluded, 123 patients (24.1%) with chronic liver disease had signs of CSPH. Patients among different SWE methods were comparable (Supplementary Table S1).

### 3.2. Prediction of Esophageal Varices

**Figure 1:**
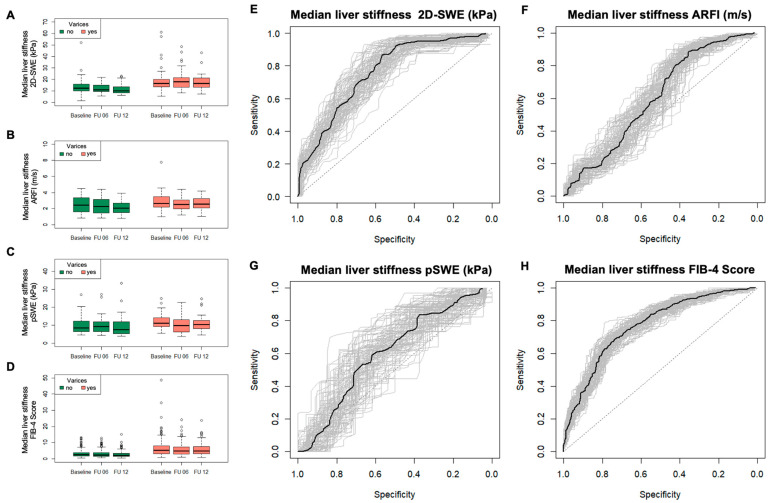
Median liver stiffness measurements for each method and FIB-4 Score at different time points (**A**–**D**), and respective ROC-curve for the prediction of esophageal varices of the pooled data over time using a bootstrap model (**E**–**H**).

Figure 1A–D display a summary of the aggregate data for the SWE and FIB-4 Score at different time points (baseline, and 6 and 12 months of follow-up). The sensitivity, specificity, NPV, and PPV of each SWE and FIB-4 Score at different time points are depicted in Supplementary Tables S2–S5. ROC-curves to visualize the performance of each method are shown in Figure 1E–H. Regarding the pooled data using the bootstrap approach, 2D-SWE had a sensitivity of 86% (95%-KI 57–95%) and specificity of 57% (95%-CI: 42–78%), with a cut-off at 12.1 kPa. PPV and NPV were 62% (95%-CI: 50–76%) and 84% (95%-CI: 69–96%), respectively. For ARFI, we calculated a sensitivity of 88% (95%-CI: 65–95%) and a specificity of 36% (95%-CI: 24–54%), and a cut-off at 1.72 m/s. PPV and NPV were 64% (95%-CI: 56–73%) and 77% (95%-CI: 63–91%), respectively. PSWE had a sensitivity of 61% (95%-CI: 33–94%) and specificity of 60% (95%-CI: 19–81%), with a cut-off at 12.1 kPa. PPV and NPV were 76% (95%-CI: 61–92%) and 73% (95%-CI: 50–100%), respectively. The FIB-4 Score had a sensitivity of 69% (95%-CI: 57–86%) and a specificity of 73% (95%-CI: 52–83%), with a cut-off at 3.5. PPV and NPV were 76% (95%-CI: 68–85%) and 72% (95%-CI: 64–81%), respectively.

### 3.3. Prediction of Clinically Significant Portal Hypertension Using Surrogate Parameters

**Figure 2:**
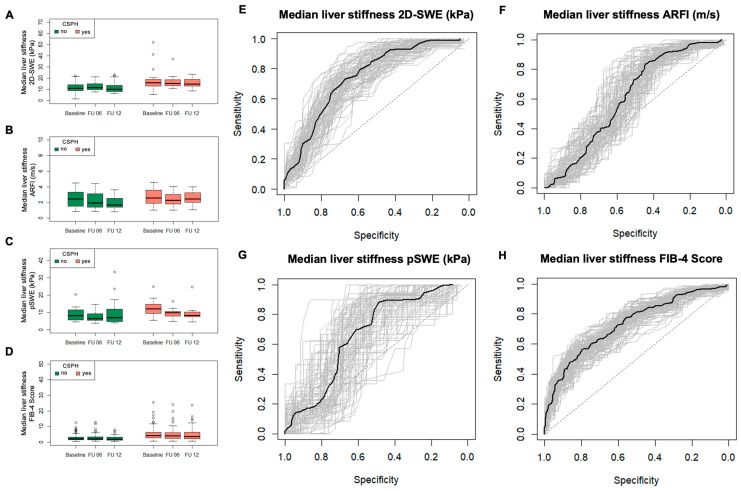
Median liver stiffness measurements for each method and FIB-4 Score at different time points (**A**–**D**), and respective ROC-curve for the prediction of clinically significant portal hypertension of the pooled data over time using surrogate parameters in a bootstrap model (**E**–**H**).

Figure 2A–D show a summary of the aggregate data for the SWE and FIB-4 Score at different time points (baseline, and 6 and 12 months of follow-up). The sensitivity, specificity, NPV, and PPV of each SWE and FIB-4 Score at different time points are depicted in Supplementary Tables S6–S9. ROC-curves to visualize the performance of each method are shown in Figure 2E–H. Regarding the pooled data, 2D-SWE had a sensitivity of 73% (95%-KI 50–96%) and a specificity of 67% (95%-CI: 38–82%), with a cut-off at 13.2 kPa. PPV and NPV were 58% (95%-CI: 40–75%) and 84% (95%-CI: 68–100%), respectively. For ARFI, we calculated a sensitivity of 84% (95%-CI: 60–95%) and a specificity of 43% (95%-CI: 27–60%), and a cut-off at 1.69 m/s. PPV and NPV were 62% (95%-CI: 51–75%) and 82% (95%-CI: 66–95%), respectively. PSWE had a sensitivity of 84% (95%-CI: 33–100%) and a specificity of 51% (95%-CI: 27–82%), with a cut-off at 7.5 kPa. PPV and NPV were 67% (95%-CI: 43–100%) and 93% (95%-CI: 76–100%), respectively. The FIB-4 Score had a sensitivity of 57% (95%-CI: 41–84%) and a specificity of 78% (95%-CI: 47–90%), with a cut-off at 3.4. PPV and NPV were 71% (95%-CI: 56–88%) and 75% (95%-CI: 66–86%), respectively.

### 3.4. Prediction of Endpoints Using a Combined Model Consisting of 2D-SWE and Platelet Count

Of all the SWE methods, 2D-SWE had the highest AUC. Recently, for patients with MASLD, a combined model using TE and platelet counts was validated and showed excellent performance in predicting CSPH using surrogate parameters [23,24]. It is now implemented in current recommendations to assess the risk of CSPH in patients with advanced chronic liver disease [25]. We therefore explored the performance of a prediction model using 2D-SWE and platelet counts to predict EV or CSPH.

**Figure 3:**
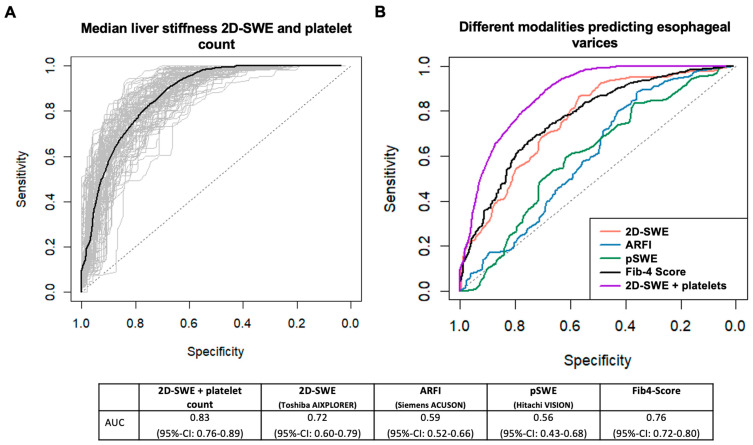
ROC-curve for the prediction of esophageal varices for the combined 2D-SWE, a platelet count model (**A**), and in comparison to the other investigated methods (**B**), with corresponding AUC values.

Using a cut-off of 42 points for the detection of EV, our model showed a sensitivity of 85% (95%-CI: 46–100%) and a specificity of 70% (95%-CI: 33–94%), with a PPV of 73% (95%-CI: 58–88%) and an NPV of 90% (95%-CI: 77–100%). Figure 3A displays the corresponding ROC curve and Figure 3B a comparison of the ROC curves of the different methods. Here, the combination of the liver stiffness 2D-SWE and platelet count was significantly better than 2D-SWE alone (difference in AUC 0.11, 95%-CI: 0.03–0.19, *p* = 0.004). A nomogram was developed, which estimates the risk of esophageal varices based on each patient’s individual data (Supplementary Figure S3A).

To predict CSPH using surrogate parameters in patients with CLD without current or previous decompensation, the combined model had a sensitivity of 70% (95%-CI: 22–100%) and a specificity 67% (95%-CI: 43–99%), with a PPV of 64% (95%-CI: 43–89%) and an NPV of 86% (95%-CI: 70–100%), using a cut-off of 56 points.

**Figure 4:**
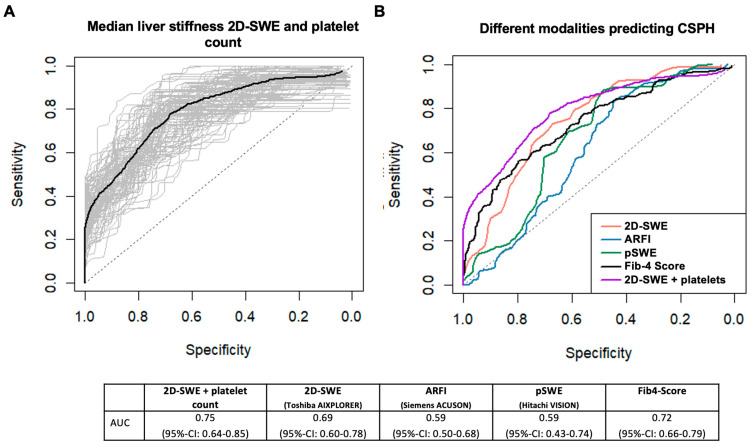
ROC-curve for the prediction of clinically significant portal hypertension for the combined 2D-SWE, a platelet count model (**A**), and in comparison to the other investigated methods (**B**), with corresponding AUC values.

Figure 4A shows the corresponding ROC curve and Figure 4B a comparison of the ROC curves of the different methods. AUC improved numerically by 0.06 (95%-CI: 0.02–0.14) in the combined model, but it was not statistically significant (*p* = 0.150). A nomogram was developed estimating the risk of CSPH using surrogate parameters based on each patient’s individual data (Supplementary Figure S3B).

**Figure 5:**
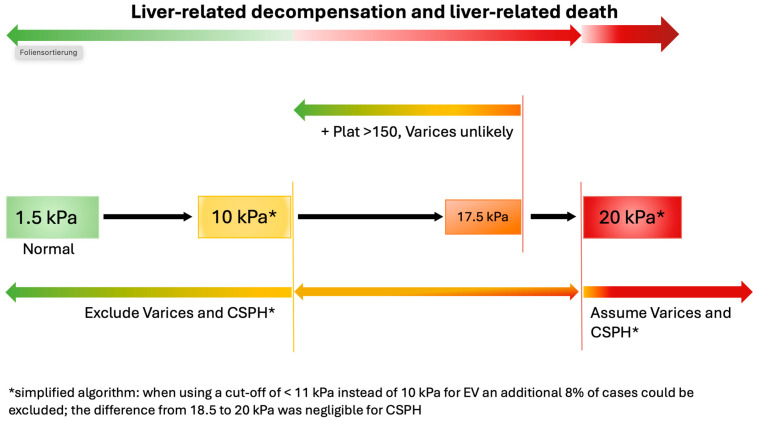
Algorithm for the non-invasive determination of esophageal varices or clinically significant portal hypertension (CSPH, defined by clinical parameters) using the platelet count and 2D-SWE liver stiffness measurement.

Finally, our aim was to develop an algorithm for the non-invasive determination of EV or CSPH using 2D-SWE and the platelet count. We calculated a cut-off to rule in or exclude both endpoints using our bootstrap models, considering a specificity and sensitivity of ≥90%, respectively. LSM > 18.5 kPa and 20 kPa were indicative of CSPH using surrogate parameters and EV, respectively, while measurements < 10 kPa ruled out CSPH and <11 kPa ruled out EV. While the addition of the platelet count to 2D-SWE was not much more useful to rule out CSPH, platelets > 150/nL in patients with an LSM of 11–17.5 kPa appear to be less frequently associated with EV. An algorithm considering these results for the daily clinical routine is depicted in Figure 5: When using a cut-off of <11 kPa instead of 10 kPA for EV, an additional 8% of cases could be excluded; the difference from 18.5 to 20 kPa was negligible for CSPH.

## 4. Discussion

In our study, we retrospectively analyzed data from a large, prospective cohort study of patients with CLD and consecutive SWE measurements. Among different SWE methods, 2D-SWE by Toshiba APLIO500 showed the best results in predicting EV in patients with CLD with and without past or present decompensation and CSPH in patients with compensated CLD using surrogate parameters. The combination model (platelet count and 2D-SWE) reliably predicted EV and CSPH in both groups and AUC increased as compared by 2D-SWE alone. Using this large real-world patient collective, an algorithm was developed to non-invasively rule in or exclude EV or CSPH by surrogate parameters in daily clinical routine, in analogy to the Baveno VII consensus approach with VCTE, which was recently validated for TE [25,29,30].

Within the last two decades, ultrasound-based liver stiffness assessments have revolutionized evaluating the fibrosis stage in patients with CLD [31]. Prior to that, liver biopsy was the standard of care to histologically categorize patients with CLD who had not yet progressed to the stage of decompensated advanced chronic liver disease [3]. The gold standard to evaluate CSPH remains the invasive measurement of the hepatic venous pressure gradient (HVPG) via hepatic vein catheterization. CSPH is defined as a HVPG ≥ 10 mmHg which is, however, impractical for daily clinical routine and hence limited to a selected group of patients and clinical trials [3,32].

VCTE is nowadays one of the most often used methods to assess liver stiffness and has extensively been studied in clinical trials [10,11,14,30]. In 2016, the authors of the ANTICIPATE study developed a reliable model to predict the risk of CSPH in patients with compensated advanced chronic liver disease by using LSM via VCTE and platelet counts [33]. This model has been validated in large multicenter trials [29,34], modified [35], and has now been implemented in the Baveno VII consensus to non-invasively diagnose or exclude CSPH in patients with CLD [25]. For clinicians, this consensus seems practical as it uses specific VCTE thresholds to include or exclude CSPH, and thereby avoids unnecessary (invasive) diagnostic procedures. However, reliable data on SWE in analogy to this tool are scarce, although SWE has been an emerging alternate technology [13,14,15]. Recently, there has been some effort to characterize cut-off values for both pSWE and 2D-SWE to predict different stages of liver disease such as CSPH [36,37]. Apart from that, different SWE methods (both pSWE and 2D-SWE) to evaluate spleen stiffness have also shown promising results to correlate with CSPH alone or to provide additional information to current liver LSM [38].

We were able to show that especially 2D-SWE in combination with platelet count showed promising results with high NPV to exclude EV or CSPH using surrogate parameters. PPV was good but less robust in our patients. Similar to the ANTICIPATE study, the predictive power of the model was improved when platelet count was included in the model, especially for the prediction of EV. We developed an algorithm showing that in patients with an LSM of <10 kPa EV or CSPH can reliably be excluded, and that patients with an LSM > 20 kPA were at a high risk of CSPH and EV. Thus, in all patients belonging to either group, screening endoscopy may be avoided. Moreover, considering the platelet count, patients with an LSM between 10 kPa and 17.5 kPa and platelets > 150/nL were unlikely to have EV. However, the addition of the platelet count was less useful in predicting CSPH, with only a marginal benefit for the combined model. Similarly to the ANTICIPATE study, AUC was >0.8 for the prediction of EV, but was lower (0.72) for CSPH. Interestingly, cut-off values in our algorithm compared to the VCTE algorithm proposed by the Baveno Consensus and others were similar though not identical: [25,35]. While our cut-off to assume CSPH was 20 kPa for 2D-SWE, it was 25 kPa for VCTE. An LSM ≤ 15 kPa plus platelets ≥ 150/nL ruled out CSPH in Baveno, while in our 2D-SWE model, the cut-off for this scenario was 17.5 kPa.

An individual patient data meta-analysis evaluated 2D-SWE to identify CSPH [22]. They reported a cut-off of <14 kPa to safely rule out CSPH, while the cut-off for ruling in CSPH was ≥32 kPa. In our study, the cut-offs were <10 kPa and >20 kPa, respectively. Of note, they included less patients in their meta-analysis (n = 328 with only 89 categorized as compensated cirrhosis), with the majority of patients presenting with ascites at the time of inclusion. Basically, all patients at this stage (ascites) have CSPH, and in daily clinical routine, no 2D-SWE is needed to risk stratify these patients as to the question whether they have CSPH or not. A small retrospective study consisting of 80 patients, evaluating pSWE (ElastPQ) and 2D-SWE (GE-LOGIQ-S8), found a comparable lower threshold to rule out EV (<10–12 kPa though platelets had to be >150/nL in both cases) [26]. Most recently, a multicenter study including 118 Chinese and Croatian patients using 2D-SWE to predict CSPH included the platelet count in analogy to the Baveno VII consensus [39]. Applying similar cut-offs for LSM (15 kPA) and platelets (≥150/nL) to exclude CSPH, the sensitivity was high (≥90%), while applying the cut-offs to include CSPH (LSM of ≥25 kPa) ruled in every patient with CSPH in their study.

The sensitivity and specificity were lower than expected for ARFI and, as reflected in earlier trials, that showed reliable results in assessing liver fibrosis [12], while pSWE by Hitachi ASCENDUS showed a high variability. For both methods, the sensitivity and NPV were good, but specificity was lacking, implicating that they were better in excluding EV or CSPH.

A limitation of our study is the retrospective analysis—though the study design and data collection had been performed prospectively. Thus, information and selection bias are possible confounders. There were no data recorded on the grading of EV, so no analysis could be made concerning varices needing treatment—as has been done in other studies [3,21]—and results of endoscopy (within the predefined timeframe) were missing in some patients. Here, the platelet count/spleen diameter ratio with a cut-off of 909 was additionally used to avoid misclassification, a parameter shown to be very robust and reliable to predict varices [28]. Unfortunately, no additional VCTE measurements were available in our study and patients were assessed by the same SWE method at baseline and during follow-up. Thus, we could not directly compare the results of VCTE and SWE or compare the performance of SWE with regards to the interindividual performance. To overcome some of these limitations, we employed the FIB-4 Score for comparison—as a well-established parameter for fibrosis assessment in patients with MASLD [6]. As no invasive HPVG measurements were available to diagnose CSPH, the endpoint was assessed using surrogate parameters of CSPH such as EV and NSBB prescription.

Taken together, our study found 2D-SWE in combination with platelet count to be a promising tool to predict EV or CSPH in patients with CLD. Data suggest it may be used to avoid screening endoscopy or guide clinicians in indicating NSBB therapy in patients with CLD, especially if VTCE is not available. Future prospective trials are needed to validate our results.

## Figures and Tables

**Table 1:** Patient characteristics of the overall cohort and the subgroup of patients with and without clinically significant portal hypertension (CSPH).

Patients’ Characteristics	All Patients (n = 511)	Patients with CSPH (n = 315)	Patients Without CSPH (n = 196)	*p* (%)
Age, y, mean (IQR)	59 (16)	59 (16)	58 (15)	0.567
Male sex, n (%)	296 (57.9)	191 (60.6)	105 (53.7)	0.14
Weight, kg, mean (IQR)	80.5 (22.0)	80.6 (22.0)	80.2 (24.0)	0.959
Etiology of cirrhosis				
Alcohol, n (%)	124 (24.3)	102 (32.4)	22 (11.2)	<0.001
Viral Hepatitis, n (%)	181 (35.4) ^#^	95 (30.2)	86 (43.9)	0.002
MASLD/MASH, n (%)	89 (17.4)	51 (16.2)	38 (19.4)	0.42
Others, n (%)	116 (22.7)	67 (21.3)	50 (25.5)	0.32
Diabetes mellitus, n (%)	321 (62.8)	220 (69.9)	101 (51.5)	<0.001
Arterial hypertension, n (%)	96 (18.8)	49 (15.8)	47 (24)	0.02
MELD Score, mean (IQR)	10(5)	11 (6)	10 (4)	0.786
Child–Pugh Score, mean; category (IQR)	6; A (1)	6; A (2)	5; A (0)	<0.001
Non-selective betablockers, n (%)	91 (17.8)	91 (28.9)	0 (0)	<0.001
FIB-4 Score, mean (SD)	4.7 (±3.9)	5.8 (±4.1)	3 (±2.7)	<0.001
Laboratory values				
Sodium, mmol/L, mean (SD)	139 (±3)	139 (±3)	139 (±2)	0.310
Creatinine, mg/dL, mean (SD)	0.9 (±0.4)	1.0 (±0.4)	0.9 (±0.5)	0.007
Albumin, g/dL, mean (SD)	4 (±0.6)	3.8 (±0.7)	4.3 (±0.5)	<0.001
Bilirubin, mg/dL, mean (SD)	1.5 (±1.6)	1.7 (±1.5)	1 (±1.8)	<0.001
Aspartate aminotransferase, U/L, mean (SD)	50 (±44)	55 (±50)	42 (±30)	<0.001
Alanine aminotransferase, U/L, mean (SD)	38 (±40)	37 (±41)	39 (±38)	0.422
Gamma glutamyltransferase, U/L, mean (SD)	127 (±177)	138 (±195)	108 (±142)	0.02
Alkaline Phosphatase, U/L, mean (SD)	116 (±78)	124 (±69)	102 (±90)	<0.001
Leucocytes. n/nL, mean (SD)	6.1 (±2.3)	5.8 (±2.5)	6.5 (±2)	<0.001
Platelets. n/nL, mean (SD)	138 (±72)	117 (±64)	173 (±70) *	<0.001
Hemoglobin. g/dL, mean (SD)	13.2 (±2.4)	12.8 (±2.6)	13.9 (±1.8)	<0.001
International normalized ratio, mean (SD)	1.2 (±0.3)	1.3 (±0.4)	1.1 (±0.3)	<0.001
Endoscopy within 24 months from baseline, n (%)	369 (72.2)	272 (86.3)	97 (49.5) *	<0.001
Ascites, n (%)	79(15.5)	79 (25.1)	0 (0)	<0.001
Esophageal Varices, n (%)	239 (46.8)	239 (75.9)	0 (0)	<0.001
Platelet/spleen ratio, mean (SD)	1114 (±698)	905 (±615)	1447 (±696)	<0.001

^#^ 43 patients with chronic hepatitis B virus infection, all on antiviral therapy, 103 patients with sustained virologic response after hepatitis C virus infection treatment, and 35 patients with chronic hepatitis C. * The number of patients with relevant thrombocytopenia was small in this group. The majority of patients with moderate or severe thrombocytopenia had a recent endoscopy without signs of esophageal or fundus varices, without portal hypertensive gastropathy, and with no signs of ascites during ultrasound examination. In 10 patients with moderate to severe thrombocytopenia, no endoscopy was performed. Here, in 7 out of 10 patients, the spleen size was small, resulting in a platelet/spleen ratio of >909 which makes varices relatively unlikely [28] (thrombocytopenia possibly related to other causes, i.e., bi- or pancytopenia or other bone marrow disorders). In the remaining 3 patients, no spleen size was documented, and no endoscopy was performed.

## Data Availability

The data presented in this study are available on requenst from the corresponding author due to local study restrictions.

## Supplementary Materials

The following supporting information can be downloaded at https://www.mdpi.com/article/10.3390/jcm13247719/s1, Figure S1: Flowchart of patients included in this study; Figure S2 Different SWE methods during examination; Figure S3: Nomograms estimating the risk of esophageal varices (A) and clinically significant portal hypertension (B) based on each patient’s individual data; Table S1: Comparison of baseline characteristics according to the different SWE methods used; Table S2: Sensitivity, specificity, negative and positive predictive value of 2D-SWE by Toshiba APLIO500 to predict the presence of esophageal varices. Table S3: Sensitivity, specificity, negative and positive predictive value of ARFI by Siemens ACUSON to predict the presence of esophageal varices. Table S4: Sensitivity, specificity, negative and positive predictive value of pSWE by Hitachi VISION to predict the presence of esophageal varices. Table S5: Sensitivity, specificity, negative and positive predictive value of FIB4-Score to predict the presence of esophageal varices. Table S6: Sensitivity, specificity, negative and positive predictive value of 2D-SWE by Toshiba APLIO500 to predict clinically significant portal hypertension. Table S7: Sensitivity, specificity, negative and positive predictive value of ARFI by Siemens ACUSON to predict clinically significant portal hypertension. Table S8: Sensitivity, specificity, negative and positive predictive value of pSWE by Hitachi VISION to predict clinically significant portal hypertension. Table S9: Sensitivity, specificity, negative and positive predictive value of FIB4-Score to predict clinically significant portal hypertension.

## Abbreviations

aCLD, advanced CLD; ARFI, acoustic radiation force impulse imaging; AUC, area under the curve; CI, confidence intervals; CLD, chronic liver disease; CSPH, clinically significant portal hypertension; EV, esophageal varices; LSM, liver stiffness measurements; NPV, negative predictive value; NSBB, non-selective beta-blockers; pSWE, point shear-wave elastography; PPV positive predictive value; ROC, receiver operating characteristic; SWE, shear-wave elastography; 2D-SWE, two-dimensional SWE.
