# Supplementary material for: Two-Dimensional and Point Shear-Wave Elastography to Predict Esophageal Varices and Clinically Significant Portal Hypertension in Patients with Chronic Liver Disease
Source: J Clin Med. 2024 Dec 18;13(24):7719. doi: 10.3390/jcm13247719 (PMC11676802; doi:10.3390/jcm13247719)
Supplement: Supplementary file 1 [file jcm-13-07719-s001.zip › jcm-3311063-supplementary.pdf]

Shear-Wave Elastographie to Predict Esophageal Varices and Clinically Significant Portal Hypertension in Patients with Chronic Liver Disease

Myriam W. Heilani[1], Max Bolender[1], Victoria T. Mücke[1], Katharina M. Schwarzkopf[1], Alica Kubesch, Nada Abedin[1], Georg Dultz[1], Stefan Zeuzem[1], Christoph Welsch[1], Mireen Friedrich-Rust[1], Jörg Bojunga[1], Eva Herrmann[2], Marcus M. Mücke[1]

Supplementary Material

Affiliations:

[1]Medical Clinic 1, University Hospital, Goethe University Frankfurt, Frankfurt am Main, Germany

[2]Institute of Biostatistics and Mathematical Modeling, Goethe University Frankfurt, Frankfurt am Main, Germany

[*]**Corresponding author, current address:**

PD Dr. med. Marcus M. Mücke, Medical Clinic 1, University Hospital, Goethe-University Frankfurt, Frankfurt am Main, Germany

Email: MarcusMaximilian.Muecke@unimedizin-ffm.de

Tel: +49 69 6301 5122

SUPPLEMENTARY METHODS

ARFI – Siemens ACUSON S 2000

ARFI imaging using a Siemens ACUSON S2000 (Virtual Touch Tissue Quantification, Erlangen, Germany) was performed with a 4C1 transducer targeting an anatomic region with a region of interest (ROI) cursor while performing real-time B-mode imaging. There was no additional modification to the manufacturer's standard SWE measurement configuration. Briefly, short duration acoustic pulses (fixed transmission frequency of 2.67 MHz) lead to localized displacements in tissue and produced mechanical excitation at the standardized ROI box. Shear wave propagation was assessed using ultrasonic, correlation-based methods.[1] The maximum displacement was estimated laterally adjacent to the single push beam. The time-to-peak displacement at each location was measured and the shear wave speed of the tissue was reconstructed (ROI: 10 x 5mm, central window for shear velocity measurement 5 x 4 mm). The shear wave propagation velocity was proportional to the square root of tissue elasticity.[1] Measurements were expressed in meters per second. See also Supplementary Figure 3A.

2D-SWE Toshiba Aplio500

2D-SWE was performed with the Toshiba AplioTM500 ultrasound system (Toshiba Medical Systems Corporation, Otawara, Japan, CE certification according to the Class II a of Medical Devices Act) with a 6C1 curvilinear transducer, the same probe used for B-mode examination while performing real-time B-mode imaging. For 2D-SWE, the assessment of stiffness was based on the generation of short shear wave pulses focused in a selected tissue area. The speed of shear wave propagation was measured using Doppler technology. The elastogram, a color-coded, two-dimensional real-time map (2,5 x 3 cm with a 10 mm circle ROI - presents softer tissue in blue and harder tissue in red) of tissue elasticity was displayed and integrated in the real-time B-mode image.[2] There was no additional modification to the manufacturers' standard SWE measurement configuration (shear wave frequency 2.2 MHz, tracking frequency 0). See also Supplementary Figure 3A.

pSWE Hitachi VISION

The third SWE evaluation was performed with the Hitachi VISION system (Hitachi HI Vision, Hitachi Medical Corporation, Japan) using the C715 5-1(R50) probe. There was no additional modification to the manufacturer's standard SWE measurement configuration. Briefly, an anatomic region with a region of interest (ROI, 1,5 x 1 cm) cursor was targeted while performing

real-time B-mode imaging (measurement presets in alignment with the manufacturers' instruction). Of note, here with one LSM several push track sequences were delivered and the shear wave velocity was acquired in multiple positions, at different depths, inside the ROI automatically within a short time (<1 second). For each measurement the system displayed a histogram with the distribution of the multiple velocity measurements including the median velocity in m/s and kPa. See also Supplementary Figure 3C.

SUPPLEMENTARY TABLES

Table 1. Comparison of baseline characteristics according to different SWE methods used.

**Patients' characteristics**	**1) Patients with ARFI (n=255)**	**2) Patients with 2D-SWE (n=143)**	**3) Patients with pSWE (n=113)**	**P**
Age, y, mean (IQR)	59 (17)	57 (16)	58 (14)	n.s.*
Male sex, n (%)	140 (54.9)	83 (58.0)	73 (64.6)	n.s.*
weight, kg, mean (IQR)	80.4 (17.8)	83.7 (22.0)	79.0 (20.0)	n.s.*
Etiology of cirrhosis				
Alcohol, n (%)	64 (25.1)	31 (21.7)	29 (25.6)	n.s.*
Viral Hepatitis, n (%)	90 (35.3)	49 (34.3)	42 (37.2)	n.s.*
MASLD/MASH, n (%)	36 (14.1)	32 (22.4)	21 (18.6)	n.s.*
Other, n (%)	65 (25.5)	31 (21.7)	20 (17.7)	n.s.*
MELD-Score at admission, mean (IQR)	10 (5)	10 (5)	11 (6)	n.s.*
Child-Pugh-Score at admission, mean (IQR)	6 (2)	6 (1)	6 (1)	n.s.*
Fib4-Score, mean (SD)	4.5 (3.2)	4.3 (±4.0)	4.8 (±3.8)	n.s.*
Laboratory values				
Sodium, mmol/l, mean (SD)	139 (±3)	139 (±3)	139 (±3)	n.s.*
Kreatinin, mg/dl, mean (SD)	0.9 (±0,4)	0.9 (±0.3)	0,9 (±0.3)	n.s.*
Albumin, g/dl, mean (SD)	4.1 (±0,6)	4.1 (±0,6)	4.0 (±0.6)	n.s.*
Bilirubin, mg/dl, mean (SD)	1,3 (±1,1)	1,4 (±1.3)	1.6 (±1.4)	n.s.*
Platelets, n/nl, mean (SD)	137 (±67)	147 (±72)	141 (±76)	n.s.*
Ascites, n (%)	38 (14.9)	23 (16.0)	18 (15.9)	n.s.*
Esophageal Varices, n (%)	123 (48.2)	62 (43.4)	54 (47.8)	n.s.*
Platelet-to-spleen ratio, mean, (SD)	1117 (±705)	1145 (±706)	1116 (±706)	n.s.*

*not significant with any comparison (1 vs. 2, 1 vs. 3 and 2 vs. 3).

Table 2. Sensitivity, specificity, negative and positive predictive value of 2D-SWE by Toshiba APLIO500 to predict the presence of esophageal varices.

	PPV	NPV	Sensitivity	Specificity	Cut off (kPa)
Baseline	0,62	0,84	0,89	0,5	12,05
FU6	0,61	0,91	0,94	0,51	10,95
FU12	0,58	0,92	0,9	0,63	12,25

Abbreviation: PPV, positive predictive value; NPV, negative predictive value; FU: follow-up; kPa, kilopascal

Table 3. Sensitivity, specificity, negative and positive predictive value of ARFI by Siemens ACUSON to predict the presence of esophageal varices.

	PPV	NPV	Sensitivity	Specificity	Cut off (m/s)
Baseline	0,59	0,74	0,9	0,31	1,71
FU6	0,56	0,75	0,88	0,35	1,68
FU12	0,64	0,75	0,86	0,48	1,97

Abbreviation: PPV, positive predictive value; NPV, negative predictive value; FU: follow-up; m/s, meters per second

Table 4. Sensitivity, specificity, negative and positive predictive value of pSWE by Hitachi VISION to predict the presence of esophageal varices.

	PPV	NPV	Sensitivity	Specificity	Cut off (kPa)
Baseline	0,7	0,64	0,77	0,55	9,09
FU6	0,76	0,44	0,56	0,67	9,68
FU12	0,61	0,77	0,84	0,5	7,39

Abbreviation: PPV, positive predictive value; NPV, negative predictive value; FU: follow-up; kPa, kilopascal

Table 5. Sensitivity, specificity, negative and positive predictive value of the Fib4-Score to predict the presence of esophageal varices.

	PPV	NPV	Sensitivity	Specificity	Cut off
Baseline	0,77	0,68	0,66	0,79	4,12
FU6	0,74	0,68	0,67	0,75	3,88
FU12	0,75	0,72	0,74	0,73	3,19

Abbreviation: PPV, positive predictive value; NPV, negative predictive value; FU: follow-up;

Table 6. Sensitivity, specificity, negative and positive predictive value of 2D-SWE by Toshiba APLIO500 to predict the presence of clinically significant portal hypertension.

	PPV	NPV	Sensitivity	Specificity	Cut off (kPa)
Baseline	0,6	0,81	0,75	0,68	14,1
FU6	0,52	0,93	0,94	0,46	10,75
FU12	0,53	0,86	0,73	0,72	13,25

Abbreviation: PPV, positive predictive value; NPV, negative predictive value; FU: follow-up; kPa, kilopascal

Table 7. Sensitivity, specificity, negative and positive predictive value of ARFI by Siemens ACUSON to predict the presence of clinically significant portal hypertension.

	PPV	NPV	Sensitivity	Specificity	Cut off (m/s)
Baseline	0,56	0,81	0,9	0,38	1,68
FU6	0,55	0,83	0,91	0,37	1,49
FU12	0,61	0,77	0,85	0,49	1,68

Abbreviation: PPV, positive predictive value; NPV, negative predictive value; FU: follow-up; m/s, meters per second

Table 8. Sensitivity, specificity, negative and positive predictive value of pSWE by Hitachi VISION to predict the presence of clinically significant portal hypertension.

	PPV	NPV	Sensitivity	Specificity	Cut off (kPa)
Baseline	0,6	0,89	0,86	0,67	9,09
FU6	0,75	0,77	0,6	0,87	9,55
FU12	0,53	0,82	0,8	0,56	7,39

Abbreviation: PPV, positive predictive value; NPV, negative predictive value; FU: follow-up; kPa, kilopascal

Table 9. Sensitivity, specificity, negative and positive predictive value of pSWE by Hitachi VISION to predict the presence of clinically significant portal hypertension.

	PPV	NPV	Sensitivity	Specificity	Cut off
Baseline	0,68	0,75	0,67	0,76	3,16
FU6	0,58	0,78	0,79	0,55	2,27
FU12	0,7	0,67	0,54	0,8	3,46

Abbreviation: PPV, positive predictive value; NPV, negative predictive value; FU: follow-up;

Supplementary Figure 1. Flowchart of patients included in this study.

Abbreviation: CSPH, clinically significant portal hypertension; LD, liver disease; SWE, shear-wave elastography.

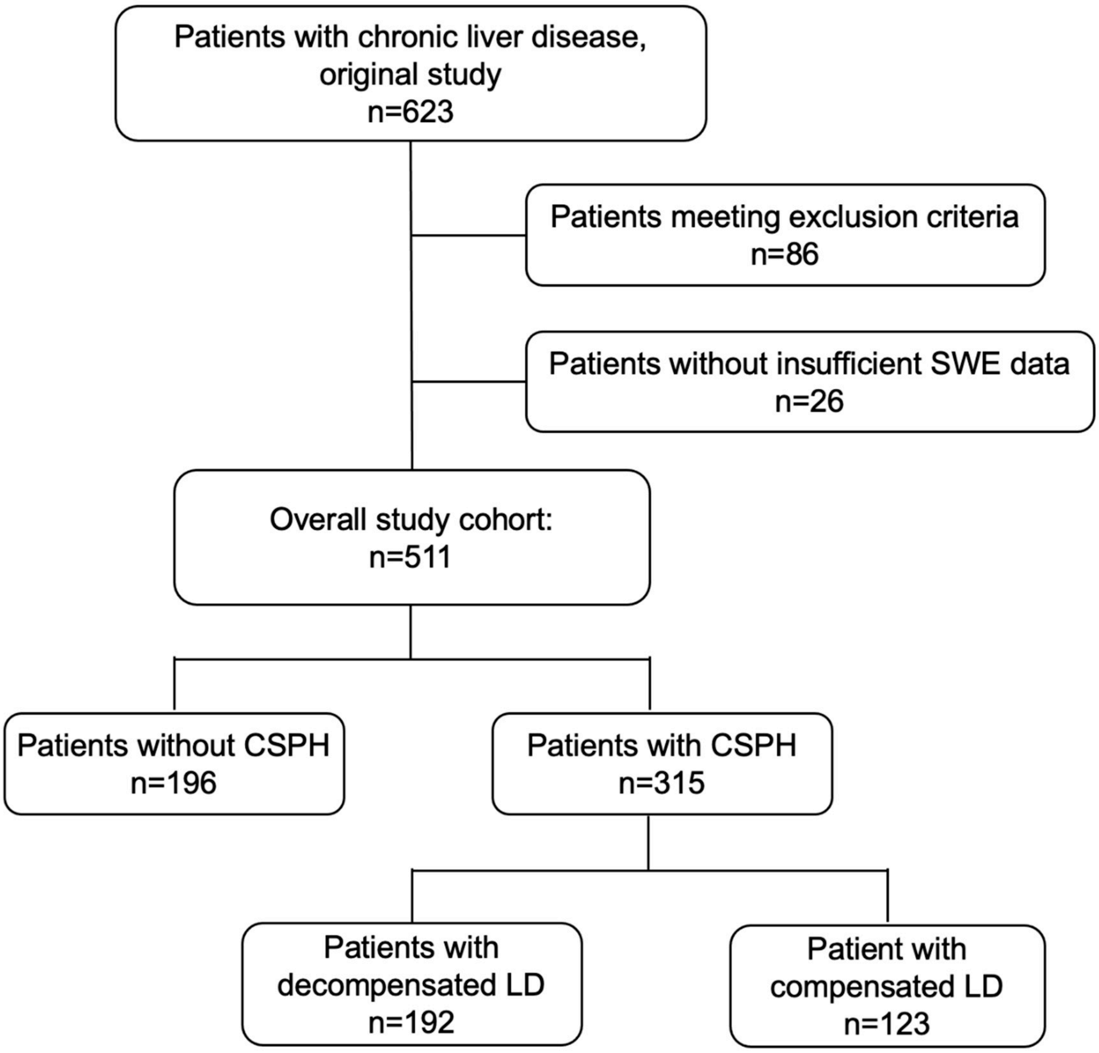

Supplementary Figure 2. Different SWE methods during examination

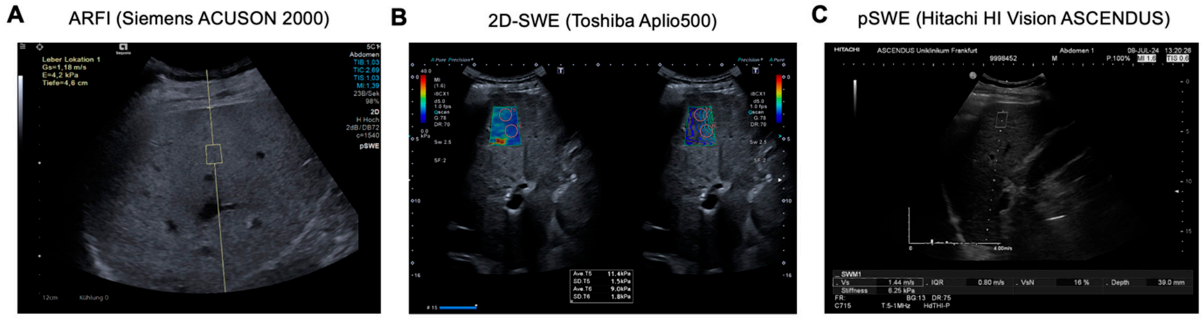

Supplementary Figure 3. Nomograms estimating the risk of esophageal varices (A) and clinically significant portal hypertension (CSPH, B) based on each patient's individual data.

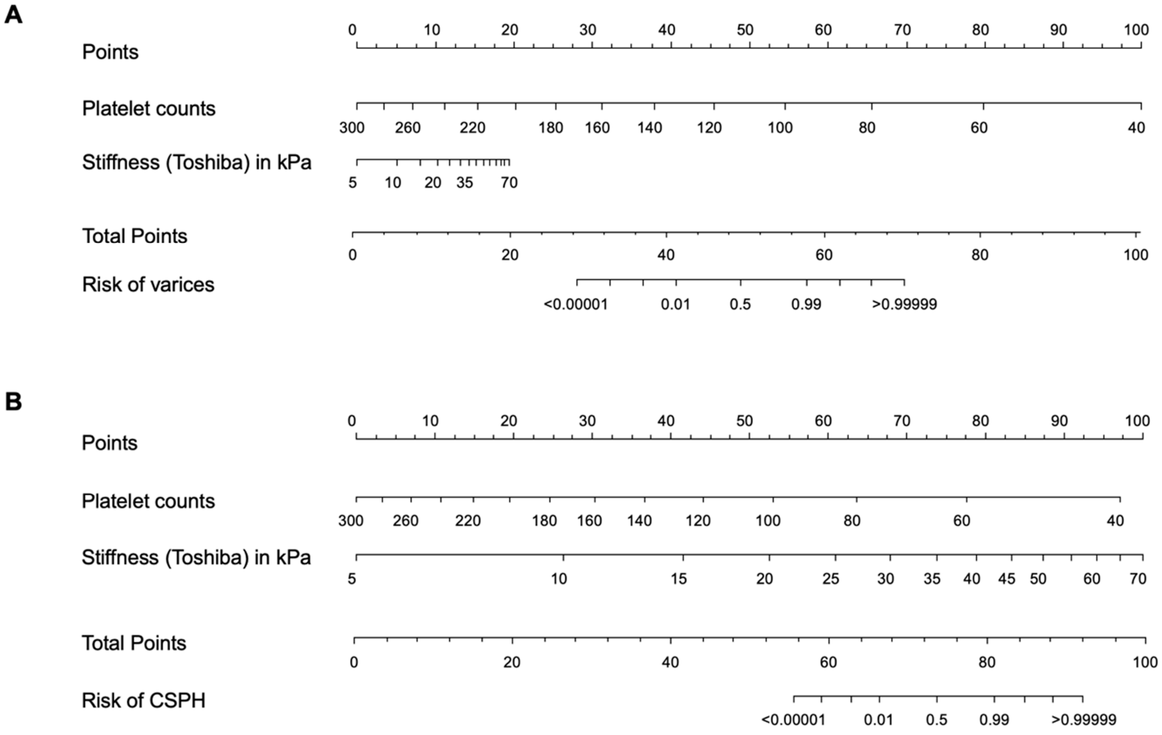

REFERENCES

1. Palmeri ML, Wang MH, Dahl JJ, Frinkley KD, Nightingale KR. Quantifying hepatic shear modulus in vivo using acoustic radiation force. *Ultrasound Med Biol* 2008;**34**(4):546-58.
2. Garra BS. Elastography: history, principles, and technique comparison. *Abdom Imaging* 2015;**40**(4):680-97.
